# How flow and mindfulness interact with each other in mindfulness-based augmented reality mandala coloring activities

**DOI:** 10.3389/fpsyg.2023.1301531

**Published:** 2024-01-08

**Authors:** Hao Chen, Chao Liu, Ayuan Zhang, Wen-Qian Lu, Kan Wu, Wen-Ko Chiou

**Affiliations:** ^1^School of Film Television & Communication, Xiamen University of Technology, Xiamen, China; ^2^Business Analytics Research Center, Chang Gung University, Taoyuan, Taiwan; ^3^School of Journalism and Communication, Huaqiao University, Xiamen, China; ^4^Teachers College, Beijing Union University, Beijing, China; ^5^College of Mechanical Engineering and Automation, Huaqiao University, Xiamen, China; ^6^Department of Industrial Engineering and Management, Ming Chi University of Technology, New Taipei, Taiwan; ^7^Department of Psychiatry, Chang Gung Memorial Hospital, Taoyuan, Taiwan; ^8^Department of Industrial Design, Chang Gung University, Taoyuan, Taiwan

**Keywords:** AR mandala coloring, mindfulness, flow, teamwork, positive psychology

## Abstract

**Introduction:**

This study explores the effects of different types of augmented reality (AR) mandala coloring activities on mindfulness and flow in college students.

**Methods:**

A total of 76 college students participated in the study and were divided into two groups based on their drawing skills: the high-skilled group (*n* = 38) and the low-skilled group (*n* = 38). With the help of AR technology, two groups of subjects carried out three mandala coloring experiments with structured mandala, free mandala and cooperative mandala in order. The measurement evaluation in the experimental program included a pre-test before all the experiments (Time 0) and each post-test after three mandala coloring activities (Time 1, Time 2 and Time 3). The balance dimensions of flow and challenge skills of the two groups were measured.

**Results:**

ANOVA results showed that a single 30-min short-term datura coloring activity did not significantly improve mindfulness (*f* = 2.339, *p* = 0.074, *η*^2^ = 0.031), but did significantly improve flow (*f* = 11.918, *p* = < 0.001, *η*^2^ = 0.139). Linear regression results found positive correlations between mindfulness and certain dimensions of flow (e.g., focus on a task, unambiguous feedback, sense of control, challenge -- skill balance, and automatic experience). Mindfulness was also found to be negatively correlated with the loss of the self-conscious component of flow. We also found that the free mandala was quite challenging for subjects in the low-skill group, while the teamwork in the cooperative mandala helped them overcome difficulties and cope with challenges.

**Discussion:**

Flow can be quickly and effectively improved through short AR mandala coloring exercises. The contribution of this study is to provide inspiration and reference for further exploring how AR mandola coloring can improve subjects’ mental state and promote the perfection and development of positive psychological mechanism.

## 1 Introduction

Mandala coloring is considered as a mindfulness-based activity that relieves negative emotional effects, especially anxiety, and has received increasingly widespread attention in the field of research (Carsley and Heath, 2018). Mandala coloring is an immersive and mindful artistic creation activity, widely accepted and used by art education and mass media due to its simple operation and ease of implementation (Liu et al., 2020a). Mindfulness-based coloring activities, such as mandala coloring, have been introduced into schools, homes, and workplaces in recent years to help people cope with worry and stress. Mandala painting is considered as a mindfulness-based art practice that combines the advantages of mindful meditation with artistic creation (Ozturk and Toruner, 2022). When performing mandala coloring activities, individuals are very focused on the present moment, specifically the individual’s attention is focused on the activity at hand, so this kind of creative practice is considered to be based on mindfulness (Respini et al., 2018).

With the rapid development of digital technology and the popularity of digital products, there have been many forms of using digital technology to assist traditional art creation in recent years, such as using augmented reality (AR) technology to do mandala coloring. This creative training has resulted in a number of AR coloring applications that have been used in research and teaching practices. Lee studied the application of AR coloring software in the treatment of autistic children and found that it played a positive role (Lee, 2019). In daily life, Mandala coloring spreads rapidly through mobile terminals such as mobile phones and tablets in the form of applications to meet people’s needs for improving mental health (Dauden Roquet and Sas, 2019). For digital natives growing up in the digital era, AR coloring has more attractive advantages than traditional coloring books, which is a trend worthy of attention from researchers and educators (Lee, 2019).

Mindfulness is a state of consciousness achieved by actively and nonjudgmentally paying attention to the current moment (Kabat-Zinn, 1990; Liu et al., 2022a). In addition, Junca-Silva et al. (2023) also proposed that mindfulness is a behavior for people to manage their attention, to achieve the goal of not actively describing the current scenario in this process. Both of these statements fit well with the concept of mindfulness activities, which uses non-judgmental forms to promote concentration and consciousness of the present events. Flow is a state of complete concentration, when the individual’s actions and attention are integrated, and the self-skills and task challenges match each other, resulting in high immersion in the task at hand and loss of self-awareness and perception of time (Csikszentmihalyi, 2002). Flow appears to be a subjective state of mind that happens when a person is completely engrossed in something and forgets about everything else (Bassi et al., 2022). There are some similarities between mindfulness and flow. Both emphasize the value of living in the moment and participating in intrinsically beautiful things, so as to relieve and get rid of anxiety, depression, worry and other negative emotions (Nien et al., 2018). In addition, both are regarded as signs of good mental health (Ovington et al., 2018). Mindfulness is closely related to flow state, and mindfulness may provide the basis for flow state (Chen et al., 2019). Flow requires being in the present moment and subconsciously focusing on an activity. Therefore, many scholars believe that focusing on the present moment is an effective way to experience flow (Chen et al., 2022a). Csikszentmihalyi (2002) describes the flow experience as “achieved through unusually intense concentration of attention within a limited area of stimulation.” Mindfulness and flow both rely heavily on awareness of the present moment (Ning et al., 2022). Previous studies have found a strong link between mindfulness and flow state, mindfulness may act as a trigger for flow (Ovington et al., 2018). Deng et al. (2022) found that flow was positively correlated with mindfulness. Some evidence showed that mindfulness plays a causal role in the mindfulness-flow relationship comes from studies of athletes, who suggest that mindfulness can benefit flow experience of athletes (Sparks and Ring, 2022). According to Liu et al. (2021), athletes who participated in mindfulness training programs reported more flow experience than those who did not. According to Hill et al. (2021), athletes with higher mindfulness ratings are also more likely to experience flow. Furthermore, in the flow experience experiment, excellent swimmers described a high level of awareness and acceptance of physiological sensations, indicating a state of mindfulness (Ning et al., 2022). The enhancement of mindfulness is closely related to the flow changes of participants, and mindfulness therapy tailored to specific athletes can effectively promote flow experience (Chen et al., 2019).

The most significant characteristic of flow is the balance between task challenges and personal skills (Jackson and Csikszentmihalyi, 1999). Based on this theory, for the sake of feeling flow, people must do something tough enough to allow them to fully utilize their talents. But this also needs to be considered, because both too much and too few challenges are inappropriate. Too much challenge will lead to irritation, and too little challenge would lead to boredom (Csikszentmihalyi, 1978). People who played ball games that needed more collaboration were more likely to experience higher joy and better flow than those who played games that required less teamwork, according to an experiment (Walker, 2010). Tse et al. (2018) looked into the possible moderating impacts of teamwork and flow propensity on the relationship between challenges and flow state. The results suggest that while challenges and flow states are adversely connected, this negative relationship may be minimized if people gather together as a team. When working resources are inadequate to satisfy job needs, one’s well-being is jeopardized, according to organizational psychology (Schaufeli and Bakker, 2004). Social support is one form of resource. When faced with greater problems, social support becomes even more crucial since it may provide as an extra resource for maintaining vitality and happy experiences when individual talents fail to fulfill the demands (van Oortmerssen et al., 2022). Perceived social support (an important element of job resources) have a positive connection with instructors’ job satisfaction in a research involving music teachers and students (Bakker, 2005). Xie (2022) found that team members who were good at regulating emotions and making suggestions more frequently reported higher group mindfulness and flow, and group mindfulness has also been found to be positively correlated with flow. Their studies originally shown that when the degree of challenge is high, teamwork may improve the flow and pleasure of the task.

### 1.1 Research purposes and hypotheses

In a word, mandala coloring has been widely concerned and praised by the society for its advantages of simplicity and easy operation (Carsley et al., 2015). Previous research has suggested that higher levels of mindfulness may help individuals feel greater flow. In the digital age, digital technology enables traditional art production, and more attention should be paid to the changes of flow and mindfulness in the AR mandala coloring environment. The purpose of this study was to explore: (1) whether AR mandala coloring contributes to mindfulness and flow; (2) Is there a connection between mindfulness and flow in AR mandala coloring creation? (3) In AR mandala coloring exercises, can teamwork improve the flow state?

Based on the flow theory that skills need to match challenges, we divided participants into two categories. The first group had a higher level of drawing skills, while the second group had a lower level of drawing skills. The AR Mandala coloring activity was set up with three task challenges of different difficulty: structured mandala, free mandala and cooperative mandala.

Structured mandala is a circular pattern made up of symmetrical forms that is claimed to help users achieve a state of mindfulness by encouraging deeper attention and engagement (Liu et al., 2019). By coloring the structural patterns inside the structured mandala, users can enter a state of intense awareness (Curry and Kasser, 2005). Coloring structural patterns has been described as a way to give users a purpose and direction, and to organize their personal experience (Carsley and Heath, 2018).

Free mandala differs from the structured mandala in that a free mandala is colored inside a completely blank circle without any initial structural pattern. Thus, the unstructured nature of free mandala coloring can be challenging for participants, as they have no reference and can only rely on their imagination and creativity to create their own patterns. Although this gives participants a free space to create, it also increases their stress, because creating patterns will have certain difficulties and may cause anxiety (Curry and Kasser, 2005).

**Figure 1:**
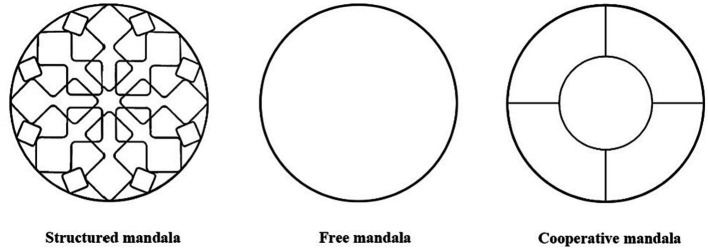
Three mandala patterns provided to the subjects in the experiment.

Collaborative mandala is a collaborative coloring process that requires multiple participants to work together through a team effort. In this study, the design of the free mandala pattern was modified to form a new cooperative mandala pattern (Figure 1). The whole pattern is composed of two concentric circles embedded together. The outer big circle is evenly divided into four parts, each participant is responsible for coloring one part, and the inner small circle is colored by four participants together. The goal of the cooperative mandala was to see if teamwork could help subjects complete difficult tasks, reduce their anxiety and enhance flow experience.

*Hypothesis 1:* Structured and collaborative mandala coloring will significantly improve participants’ mindfulness and flow in both two groups.

*Hypothesis 2:* Free mandala coloring wont significantly improve mindfulness and flow of participants in low-skill group.

*Hypothesis 3:* In the free mandala coloring, significant differences in flow and challenge-skill balance will be determined between the high-skill and low-skill groups.

## 2 Methods

### 2.1 Research procedure and design

The study used a 2 (group) × 4 (time) parallel trial design. We took a closer look at each participant’s drawing skills and specifically separated them into two groups: High-skill group (students majoring in art design or painting lovers with more than 6 months training experience in art training institutions, *n* = 38) and Low-skill group (students not majoring in art design and have no painting experience, *n* = 38). Inclusion criteria were: (1) Over the age of 18; (2) voluntarily agreed to participate in this study and sign informed consent; (3) have sufficient cognitive ability, reading and writing ability to complete the questionnaire. Exclusion criteria were: (1) color blindness; (2) taking psychotropic drugs; (3) electronic device use disorder; (4) have participated in any form of psychological intervention in the last year or currently; (5) have previous mandala coloring experience in any form. A list of codes originally labeled “Group 1” and “Group 2” was provided to participants by the group staff. The experimental intervention staff did not obtain the group list during the experimental intervention. After the data analysis was completed, the group staff informed the data analysts and the experimental intervention staff of the meaning of each group in the group list. Through this process, double blindness was achieved. Through the above experimental design, we strive to ensure the internal validity of the experiment as much as possible.

**Figure 2:**
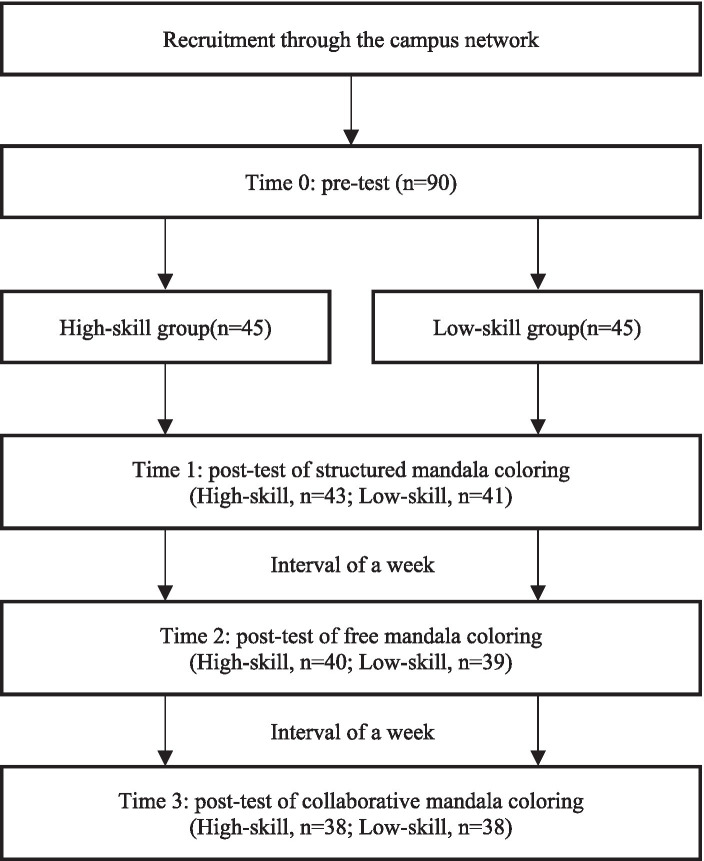
Procedure flow chart.

We conducted three trials using three forms of AR mandala coloring (structured mandala, free mandala, and cooperative mandala) on two groups (high-skill and low-skill) of participants. There was a one-week interval between the two adjacent experiments. In the first week of the structured mandala coloring experiment, participants were instructed to use the software for 30 min. Each participant was then given a form to collect demographic information, an informed consent form, and a pre-test assessment (time 0) questionnaire (SMS and SDFS-2 scales). The purpose of the pre-test is to take baseline measurements. The participants then embarked on a 30-min AR structured mandala coloring activity. When participants completed their mandala coloring, the same assessment questionnaires (SMS and SDFS-2 scales) were provided again for post-test evaluation (time 1). In the second week, both groups underwent a free mandala coloring experiment, which included 30 min of AR free mandala coloring and a post-test (time 2) at the end of the experiment. Also in the third week, the two groups of subjects carried out AR cooperative mandala coloring experiment for 30 min and post-test (time 3). Participants were allowed to unconditionally terminate the experiment and quit at any time, with the researchers protecting their privacy. Please refer to Figure 2 for the flow chart of the experimental procedure. In order to increase the participants’ motivation to participate in the experiment and reduce the attrition rate, participants who fully participate in the 3 experiments and complete the scale 4 times will be presented with a souvenir worth 50 RMB as a token of appreciation.

### 2.2 Participants

The participants were all students at a university in China. We published recruitment information through the university’s internal network and student community. AR Mandala coloring is a self-exploration and spiritual healing activity combining technology and art, which can help participants better understand themselves and relax their body and mind. Participants who were interested and eligible to participate in our study provided their registration information. Ultimately, 76 subjects were eligible to participate and complete the experiment. All of them were between the ages of 18 and 46. The demographic characteristics of the participants is shown in Table 1. The Chi-square test results in Table 1 show that there is no significant demographic difference between the two groups of subjects.

**Table 1:** Demographic characteristics of participants.

Characteristic		Total	High-skill group	Low-skill group	*χ* ^2^	*p*
Age (SD)		22.51 (5.56)	20.61 (3.15)	24.42 (6.73)		
Gender	Male	57 (75.0%)	32 (84.2%)	25 (65.8%)	3.439	0.064
	Female	19 (25.0%)	6 (15.8%)	13 (34.2%)		
Education	Undergraduate	51 (67.1%)	28 (73.7%)	23 (60.5%)	1.520	0.468
	Postgraduate	17 (22.4%)	7 (18.4%)	10 (26.3%)		
	PhD student	8 (10.5%)	3 (7.9%)	5 (13.2%)		

### 2.3 Data collection and instruments

We provided each subject a tablet computer with the AR coloring software “Pigmentation” on it. This software has a rating of 4.5 on Apple App Store, and has a large number of users worldwide. The software can identify the mandala pattern on the paper through the camera of the tablet, and then generate the corresponding three-dimensional mandala stereoscopic pattern on the screen. The mandala stereoscopic pattern can be colored by the finger and other interactive operations such as moving, rotating and scaling can be carried out. It can take photos and fill in colors, and the coloring method is as similar as possible to the traditional coloring. The difference between AR coloring and traditional coloring is that AR coloring is done using fingers on the screen of the tablet, while traditional coloring is done with colored pens on paper. Each subject will perform a pattern coloring task within the software that is consistent with the type of mandala painting proposed in the above experiments (Figure 1). Each participant was able to apply the color directly to the tablet using their fingers. After the coloring, each subject will see and interact with the AR image of their colored datura through the app.

The questionnaire used in this study consists of three parts: demographic information, State Mindfulness Scale (SMS), and Dispositional Flow Scale-2 (DFS-2). The demographic data section includes age, gender, education and drawing skills assessment. Both the SMS and DFS-2 were originally developed in English and have been translated into Chinese and have been proven to have good reliability and validity.

SMS is a frequently used mindfulness measurement tool developed by Tanay and Bernstein (2013). This is a 21-item assessment scale that tends to measure the subject’s state of mindfulness, including subscales on two dimensions: mindfulness of mind (15 items) and mindfulness of body (6 items). Each item used a 5-point Likert scale ranging from 1 (strongly disagree) to 5 (strongly agree). The scores of the scale indicate the degree of state mindfulness of the subject. The tool was highly effective in assessing subjects’ subconscious and non-judgmental perception, surpassing similar scales used to assess mindfulness and current awareness (Mantzios and Giannou, 2018). In current study, Cronbach’s alpha of SMS was 0.91. For each dimension, mindfulness of body was 0.85 and mindfulness of mind was 0.93.

DFS-2 is the most widely used flow measuring instrument developed by Jackson et al. (2008). The scale contains a total of 36 items and 9 dimensions: challenge-skill balance, merging action-awareness, clear goals, unambiguous feedback, concentration on task, sense of control, loss of self-consciousness, time transformation, and autotelic experience. Each dimension contains 4 items. All items used a 5-point Likert scale, ranging from 1 (strongly disagree) to 5 (strongly agree). The total scale score is calculated by adding the responses to all the items, ranging from 36 to 180 points, and assesses how strongly the subject experiences flow. Previous studies have shown that the Chinese translation version of DFS-2 was very effective and reliable in assessing the flow of the subjects, because its reliability and validity levels were basically the same as the original scale (Liu et al., 2012). In this study, the Cronbach’s alpha of DFS-2 was 0.89.

### 2.4 Data analysis

Based on the original data, data sorting, ANOVA and linear regression were conducted in IBM SPSS 22.0. The software mGENOVA was used for multivariate generalizability analysis. The confidence interval was set at 95% and the significance level was set at 0.05. Descriptive statistics were used to describe the distribution of subjects’ basic data, and ANOVA was used to compare the differences before and after the intervention. Linear regression was used to analyze the influence of mindfulness on flow. Multivariate generalizability analysis was used to test the reliability and validity of the scale.

## 3 Results

### 3.1 Multivariate generalizability analysis

MGA includes two processes: Generalizability Study (G Study) and Decision Study (D study). The main purpose of G Study is to confirm the measurement objectives, measurement sides and their relationships, and use the method of variance analysis or multivariate analysis of variance to decompose the variance and covariance components of various effects. On the basis of the variance component estimate obtained from the G Study, the D study estimated the relevant indicators used to evaluate the characteristics of the scale and the composite score, and evaluated the reliability of the scale measurement. In this analysis, multivariate *p* × *i* random measurement mode was selected, with participants (*p*) as the measurement target and test items (*i*) as the measurement side, p and i were assumed to be completely random and have interaction effects.

The Chinese version of SMS has 21 items, including two factors: mindfulness of mind (15 items) and mindfulness of body (6 items). According to the two-factor study design, the estimation matrix of variance and covariance components of participants (p), test items (i) and interaction effects between participants and test items (*p* × *i*) can be obtained at the G study (Table 2).

**Table 2:** The G study results of state mindfulness scale.

Effect	Two factors	
	Mindfulness of mind	Mindfulness of body
*p*	**0.427**	0.656
	0.484	**0.250**
*i*	0.023	
		0.020
*p* × *i*	0.548	
		0.590

The elements on the main diagonal (the bold values) are the variance component estimation of each effect on the corresponding factor, the elements below the diagonal are the covariance component estimation of the inter-factor, and the elements above the diagonal are the correlation coefficient estimation of the inter-factor. *P*: the variance components on the factors effect; *i*: the variance components on the items effect; *p* × *i*: the interaction effect between the factors and the items.

According to the results in Table 2, the variance component of each factor is 0.427 and 0.250 respectively, which are relatively low, indicating that the two factors have a good effect on measuring mindfulness of subjects. The covariance component on the effect of the subjects (0.484) is relatively high according to the proportion of the variance component of the factors, and the degree of correlation between the two factors is also high relatively (0.656). In addition, the variance components on the items effect (*i*) are relatively small (< 0.1), but the interaction effect between the factors and the test items (*p* × *i*) are relatively large (> 0.5), indicating that the error of each factor is small. However, whether the two factors can be combined remains to be referred to the results of the D study.

According to the variance and covariance matrix estimated by the G study, in the D study, the universe score variance and relative error variance of the subjects on the two factors can be estimated, and then the generalizability coefficient and reliability index can be obtained. The results of the D study are shown in Table 3.

**Table 3:** The D study results of state mindfulness scale.

Indicators	Mindfulness of mind	Mindfulness of body	Composite
Universe score variance	0.284	0.256	0.259
Relative error variance	0.029	0.028	0.014
Absolute error variance	0.031	0.029	0.015
Error variance for mean	0.002	0.002	0.001
Generalizability coefficient	0.907	0.902	0.948
Reliability index	0.902	0.904	0.945
Relative signal-noise ratio	9.805	9.176	18.159
Absolute signal-noise ratio	9.186	8.735	17.166

It can be seen from the D study results in Table 3 that the generalizability coefficient, reliability index and their composite score are all reach the ideal level (> 0.9). The generalizability coefficient and reliability index of the composite score are higher than the corresponding scores of each factor. And the composite error variance is lower than the error variance of each factor.

The Chinese version of DFS-2 has 36 items, including 9 factors: challenge-skill balance, merging action-awareness, clear goals, unambiguous feedback, concentration on task, sense of control, loss of self-consciousness, time transformation, and autotelic experience. Each factor contains 4 items. The estimation matrix of variance and covariance components of participants (*p*), test items (*i*) and interaction effects between participants and test items (*p* × *i*) can be obtained at the G study (Table 4).

**Table 4:** The G study results of dispositional flow scale-2.

Effect	Nine factors								
	F1	F2	F3	F4	F5	F6	F7	F8	F9
*p*	**0. 466**	0. 330	0. 241	0. 298	0. 318	0. 273	0. 344	0. 236	0. 328
	0. 454	**0.349**	0. 397	0. 306	0. 357	0. 469	0. 480	0. 321	0. 465
	0. 697	0. 317	**0. 243**	0. 402	0. 196	0. 358	0. 283	0. 318	0. 383
	0. 389	0. 717	0. 302	**0. 245**	0. 279	0. 445	0. 349	0. 159	0. 241
	0. 442	0. 350	0. 650	0. 455	**0. 481**	0. 250	0. 365	0. 388	0. 454
	0.439	0. 472	0. 326	0. 572	0. 391	**0. 228**	0. 481	0. 443	0. 331
	0. 382	0. 398	0. 397	0. 306	0. 515	0. 412	**0. 447**	0. 232	0. 290
	0.542	0. 390	0. 432	0. 637	0. 380	0.623	0. 467	**0. 251**	0. 262
	0. 374	0. 584	0. 336	0.327	0. 417	0. 319	0. 326	0. 492	**0.163**
i	0. 063								
		0. 016							
			0. 023						
				0. 031					
					0. 015				
						0. 030			
							0. 041		
								0. 012	
									0. 020
*p* × i	0. 601								
		0. 501							
			0. 548						
				0. 887					
					0. 743				
						0. 555			
							0. 609		
								0. 512	
									0. 575

The elements on the main diagonal (the bold values) are the variance component estimation of each effect on the corresponding factor, the elements below the diagonal are the covariance component estimation of the inter-factor, and the elements above the diagonal are the correlation coefficient estimation of the inter-factor. *P*: the variance components on the factors effect; *i*: the variance components on the items effect; *p* × *i*: the interaction effect between the factors and the items. F1: challenge-skill balance; F2: merging action-awareness; F3: clear goals; F4: unambiguous feedback; F5: concentration on task; F6: sense of control; F7: loss of self-consciousness; F8: time transformation; F9: autotelic experience.

As can be seen from Table 4, the variance component of each factor (the main diagonal element) is not very high (<0.5), and the degree of correlation among the factors are also low (all between 0.1 and 0.5), indicating that there is a certain independence among the factors. The interaction effect between the factors and the items (*p* × *i*) are relatively high (> 0.5), while the variance components of the items (i) are low (< 0.1). However, the covariance between the factors is relatively high (all between 0.3 and 0.8), which is a basis for the factor score to be synthesized into the total score. Whether the nine factors can be synthesized needs further reference to the results of the D study.

It can be seen from Table 5 that the generalizability coefficient and reliability index of the 9 factors all reach the acceptable level (> 0.6). The generalizability coefficient and reliability index of the composite score reach the ideal level (> 0.9), and the relative error and absolute error variance of the composite score are smaller than the corresponding results of each factor.

**Table 5:** The D study results of dispositional flow scale-2.

Indicators	F1	F2	F3	F4	F5	F6	F7	F8	F9	Composite
Universe score variance	0. 297	0. 217	0. 443	0. 545	0. 417	0. 528	0. 848	0. 643	0. 763	0. 202
Relative error variance	0. 150	0. 075	0. 137	0. 222	0. 186	0. 139	0. 152	0. 128	0. 144	0. 016
Absolute error variance	0. 166	0. 079	0. 143	0. 230	0. 189	0. 146	0. 162	0. 131	0. 149	0. 017
Error variance for mean	0. 016	0. 004	0. 006	0. 008	0. 004	0. 008	0. 011	0. 003	0. 005	0. 001
Generalizability coefficient	0. 664	0. 742	0. 764	0. 711	0. 692	0. 792	0. 848	0. 834	0. 841	0. 925
Reliability index	0. 641	0. 733	0. 756	0. 704	0. 688	0. 783	0. 839	0. 831	0. 837	0. 921
Relative signal-noise ratio	1. 977	2. 883	3. 233	2. 458	2. 245	3. 806	5. 576	5. 028	5. 304	12. 284
Absolute signal-noise ratio	1. 788	2. 742	3. 102	2. 375	2. 201	3. 614	5. 224	4. 915	5. 128	11. 733

F1: challenge-skill balance; F2: merging action-awareness; F3: clear goals; F4: unambiguous feedback; F5: concentration on task; F6: sense of control; F7: loss of self-consciousness; F8: time transformation; F9: autotelic experience.

Through the results of multivariate generalization analysis, it can be seen that the Chinese version SMS and DFS-2 scales used in this study have good reliability and validity. And the score proportion of each factor of two scales and the design of the number of items are reasonable and perfect. SMS and DFS-2 are appropriate tools for measuring mindfulness and flow, respectively.

### 3.2 ANOVA

Three 2 (group type: high-skill, low-skill) × 4 (time: time0, time1, time2, time3) ANOVAs with repeated measures were conducted on mindfulness (SMS), flow (SDFS-2), and challenge-skill balance (sub scale of SDFS-2). The descriptive statistics are shown in Table 6, and the results of ANOVA are shown in Table 7.

**Table 6:** Descriptive statistics.

Group	Measures	Mean (SD)			
		Time 0	Time1	Time 2	Time 3
High-skill	SMS	3.72 (0.55)	3.82 (0.66)	3.65 (0.56)	3.64 (0.63)
	SDFS-2	3.18 (0.61)	3.51 (0.71)	3.52 (0.51)	3.53 (0.64)
	Challenge-skill	2.90 (0.92)	3.40 (0.89)	3.61 (0.76)	3.58 (0.79)
Low-skill	SMS	3.85 (0.45)	3.80 (0.57)	3.76 (0.48)	3.70 (0.57)
	SDFS-2	3.28 (0.50)	3.45 (0.50)	3.27 (0.44)	3.59 (0.53)
	Challenge-skill	3.16 (0.95)	3.53 (0.86)	3.18 (0.73)	3.55 (0.72)

SMS, state mindfulness scale; SDFS-2, the short dispositional flow scale 2; Challenge-skill, challenge-skill balance dimension of SDFS-2; Time 0: pre-test; Time 1: post-test of structured mandala; Time 2: post-test of free mandala; Time 3: post-test of cooperative mandala.

**Table 7:** Results of ANOVA with repeated measures.

Measure	Variable	F	*p*	*η* ^2^
SMS	Time	2.339	0.074	0.031
	Group	0.469	0.495	0.006
	Time×Group	0.649	0.585	0.009
SDFS-2	Time^***^	11.918	< 0.001	0.139
	Group	0.131	0.719	0.002
	Time × Group^*^	3.755	0.012	0.048
Challenge-skill	Time^***^	8.216	< 0.001	0.100
	Group	0.011	0.918	0.000
	Time × Group^*^	3.284	0.022	0.042

SMS, state mindfulness scale; SDFS-2, the short dispositional flow scale 2; Challenge-Skill, challenge-skill balance dimension of SDFS-2; *p < 0.05; ***p < 0.001.

According to the results of ANOVA, AR mandala coloring has no significant effect on mindfulness. But it significantly increased flow. In terms of challenge and skill balance, there is a significant time group interaction effect. Hypothesis 1 is partially supported.

**Figure 3:**
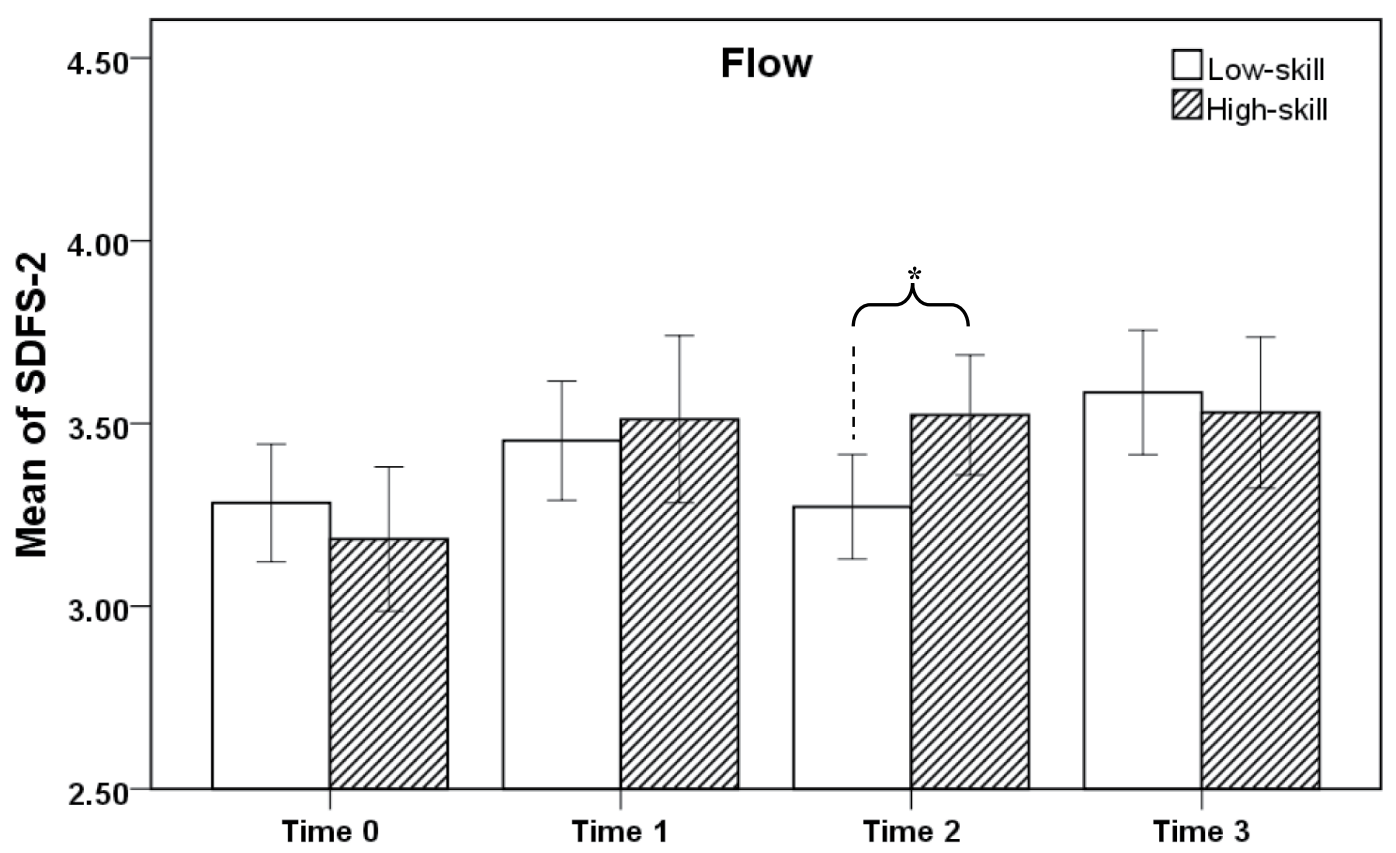
Comparison of mean SDFS-2 between the two groups. Time 0: pre-test; Time 1: post-test of structured mandala; Time 2: post-test of free mandala; Time 3: post-test of cooperative mandala; *p < 0.05.

From the results of flow, the three types of AR mandala coloring can significantly improve the flow of the high-skill group. But the free mandala did not significantly improve the flow experience of the low-skill group (Figure 3).

**Figure 4:**
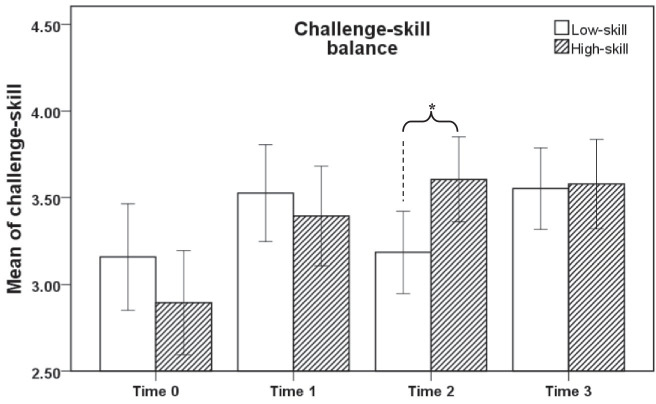
Comparison of mean challenge-skill balance between the two groups. Time 0: pre-test; Time 1: post-test of structured mandala; Time 2: post-test of free mandala; Time 3: post-test of cooperative mandala; *p < 0.05.

From the results of challenge-skill balance, the three types of AR mandala coloring can significantly improve the score of challenge-skill balance of the high-skill group. But the free mandala did not significantly improve the score of challenge-skill balance of the low-skill group (Figure 4). Hypothesis 2 is supported.

The post-paired sample *T*-test show that the flow scores of the two groups are significantly different at Time 2, *t*(74) = 2.307, *p* = 0.024. There is also a significant difference in the challenge-skill balance scores between the two groups at Time 2, *t*(74) = 2.472, *p* = 0.016. Therefore, hypothesis 3 is supported.

### 3.3 Linear regression

We identified the score of mindfulness as a dependent variable, and the independent variable was identified as 9 dimensions of flow. In order to further explore the correlation between mindfulness and flow, we applied the approach of stepwise linear regression. Table 8 summarizes these findings. The results of linear regression show that mindfulness is positively correlated with several dimensions of flow (e.g., concentration on task, unambiguous feedback, sense of control, challenge-skill balance, and autotelic experience), and negatively correlated with the dimension of loss of self-consciousness.

**Table 8:** Stepwise linear regression approach to analyze the correlation between mindfulness and flow.

Time	*R* ^2^	Predictors selected by stepwise linear regression					
Time0	0.23	Concentration on task		Unambiguous feedback			
		B (SE)	*β*	B (SE)	*β*		
		0.18 (0.07)	0.30^*^	0.17 (0.07)	0.27^*^		
Time1	0.33	Sense of control		Autotelic experience		Loss of self-consciousness	
		B (SE)	*β*	B (SE)	*β*	B (SE)	*β*
		0.29 (0.08)	0.43^***^	0.24 (0.08)	0.31^**^	−0.13 (0.05)	−0.26^*^
Time2	0.16	Unambiguous feedback		Sense of control			
		B (SE)	*β*	B (SE)	*β*		
		0.18 (0.08)	0.27^*^	0.14 (0.07)	0.23^*^		
Time3	0.46	Concentration on task		Challenge-skill balance		Unambiguous feedback	
		B (SE)	*β*	B (SE)	*β*	B (SE)	*β*
		0.27 (0.07)	0.36^***^	0.22 (0.08)	0.28^**^	0.17 (0.07)	0.24^*^

*p < 0.05; **p < 0.01; ***p < 0.001. Time 0: pre-test; Time 1: post-test of structured mandala; Time 2: post-test of free mandala; Time 3: post-test of cooperative mandala.

## 4 Discussion

### 4.1 Results and significance

Mindfulness and flow both fall under the category of positive psychology and have received a lot of attention in the research field (Liu et al., 2020c, 2022b). Previous research has found that some of the key characteristics of flow are associated with higher levels of mindfulness, such as challenge-skill balance, clear goals, focus, clear feedback, a sense of control, and a loss of self-awareness (Ovington et al., 2018). According to the linear regression results of this study, mindfulness is positively correlated with flow characteristics such as challenge-skill balance, task focus, explicit feedback, and a sense of control.

Mindfulness, on the other hand, is thought to be negatively associated with the loss of this single-minded feature of self-awareness (Liu et al., 2020b; Chen et al., 2021). Improving a person’s ability to maintain awareness while doing something may hinder their immersion in the process. Mindfulness and flow may seem similar at first glance, both involve effective and high-quality use of the mind and a heightened focus on the present moment, and both are considered indicators of good mental health and wellness. Therefore, many studies believe that improving people’s mindfulness will also help them feel flow. However, mindfulness and flow are not identical in their respective subdimensions. Mindfulness is often described as a form of mental discipline that requires self-control and a commitment to maintaining reflective awareness of each moment. Flow experience means that people forget about the passage of time and loss of self-consciousness when they are focused on something (Chen et al., 2022b). Flow is an engaging state of mind that occurs naturally when a person is immersed in the task at hand. Mindfulness seems to reduce absorption, which is an important component of flow (Sheldon et al., 2015; Liu et al., 2022c). According to the famous metaphor of “stream of consciousness” of James (1985), mindfulness seems to require a person to stand on land beside a stream and observe the stream, but be careful not to fall into it. Flow is someone who jumps into a stream, feels the stream, and responds positively to challenges.

According to the results of data analysis, none of the three types of AR mandala painting significantly improved mindfulness. Although mandala coloring is thought to be associated with mindfulness, it has not been definitively confirmed whether mandala coloring can actually enhance mindfulness (Mantzios and Giannou, 2018). There is little evidence that datura coloring directly enhances mindfulness, and the results are mixed (Carsley et al., 2015). Carsley and Heath (2018) confirmed the advantages of mandala coloring activities in their analysis of 152 children. They found that mandala coloring reduced test anxiety in children more significantly than other free drawing activities, with test anxiety significantly decreased and concentration significantly improved. Mantzios and Giannou (2018) aimed to determine whether completing mandala coloring improved the level of mindfulness in 88 college students. According to their results, there was no substantial difference in reducing anxiety between mandala coloring and free painting.

The reason is that the improvement of mindfulness requires teaching long-term persistence and systematic training (Liu et al., 2022d). MBSR (Mindfulness Based Stress Reduction) is an eight-week mindfulness and emotion management training program that is widely considered to be beneficial for enhancing mindfulness (Kabat-Zinn, 1990). Perhaps 30 min of mandala coloring course is not enough to cultivate mindfulness, mindfulness training needs a certain amount of time and method guidance to achieve a better effect. In addition, there are differences between AR coloring and traditional coloring in coloring accuracy and mapping methods, which are believed to affect the state of mindfulness (Daudén Roquet et al., 2021). However, it is undeniable that datura is gaining traction in popular culture, with subjects showing more interest and concern for AR coloring itself. From the results, it can be seen that flow improved significantly after the experimental intervention, while mindfulness did not change significantly. Flow, after all, is defined by a lack of self-awareness and total immersion in a task, but mindfulness requires constant self-awareness.

The results showed that all three types of AR mandala coloring significantly improved the flow scores of the high-skill group. On the other hand, the flow scores of the low-skill group did not improve significantly in the free mandala coloring experiment. Similar results were found for the challenge-skill balance dimension. This finding suggests that the free mandala is difficult for low-skilled subjects, but that the teamwork factor in cooperative mandala coloring may help them better cope with this challenge. Tse et al. (2018) studied the correlation between collaboration and flow propensity to challenge and flow states. Their findings suggest that although there is a negative correlation between flow and challenge, this negative correlation can be improved by people engaging in teamwork. Teamwork helps mediate the correlation between challenge and flow states. Individuals with higher skill levels usually have higher flow proneness, and they are more inclined to seek challenging jobs to maintain a higher level of flow experience (Sinnott et al., 2020). They are also confident in their ability to deal with these issues, and these people are attracted to activities that bring about a flow experience. While it is commonly believed that those who complete extremely challenging tasks may feel less positive emotions (and are more likely to spot dilemmas), participants with high levels of flow proneness tend to be more stable in such situations. In this case, actively responding to challenges will produce stronger positive emotions. On the contrary, low-skilled individuals will rely more on the external atmosphere or the motivation of others when undertaking challenging tasks (Feng, 2022). Because highly interdependent collaboration among team members creates a higher happiness and flow experience for the entire team. Therefore, promoting collaboration among team members seems to be an important aspect of improving the flow experience and thus helping the less skilled to meet the challenge flow.

### 4.2 Research limitations and future studies

Here are some limitations of this study: (1) The study’s participants were mostly college students and mostly female. Therefore, the results of this study should be interpreted and generalized with caution. Whether the results of this study can be applied to other different types of sample groups needs to be further tested. (2) The intervention time of AR mandala coloring activity used in this study was short, only 30 min each time. Currently, only a few studies have focused on the effects of long-term mandala coloring practices, and the results of these studies may also vary. (3) There is a difference in the coloring operation mode between the AR mandala used in the experiment and the traditional mandala, which may also affect the psychological state and experience of the participants. (4) The order of trial conditions in this study was fixed, rather than random or counterbalanced. So, the effects over time may be confounded with a practice effect. In order to avoid this, we arranged a one-week interval between each experiment to mitigate the practice effect. (5) Since taking into account the participants’ acceptance of the number of tests, the pre-test was performed only once at time 0, rather than redone for each experiment. So, there may have been changes in the baseline mindfulness and flow across the 3 weeks of testing.

Future studies need to use larger, more diverse samples. Although the results of the data analysis in this study showed that gender had no significant effect on the results. Some previous studies have also confirmed that gender has no significant effect on mindfulness (Liu et al., 2022e) and flow (Tse et al., 2018). Nevertheless, the proportion of various demographic characteristics of participants within and between groups should be maintained to the extent practicable, and the influence of demographic variables on experimental results must be minimized. Efforts are made to reduce the threat of each variable to the internal and external validity of the experiment. Future research should look at the longer-term effects of AR mandala coloring activities on mindfulness and flow, as some of the interactional relationships between mindfulness and flow may not be immediately apparent, but gradually emerge and build up over time. Future studies will also consider comparing the effects of AR mandala coloring with other mindfulness-based activities on subjects.

## 5 Conclusion

In a short period of time, the AR mandola coloring activity has no promoting effect on the state of the mindfulness, but the improvement in the state of the flow is extremely obvious. Mindfulness was positively correlated with several dimensions of flow (e.g., concentration on task, unambiguous feedback, sense of control, challenge-skill balance, and autotelic experience). On the other hand, mindfulness was found to have a negative relationship with the loss of self-consciousness dimension. Respondents in the low-skill group may find free mandala difficult, however cooperative mandala might assist them get over this challenge. The contribution of this study is to provide inspiration and reference for further exploring how AR mandola coloring can improve subjects’ mental state and promote the perfection and development of positive psychological mechanism.

## Data availability statement

The original contributions presented in the study are included in the article/supplementary material, further inquiries can be directed to the corresponding author.

## Ethics statement

This study was reviewed and approved by the academic committee of Chang Gung University. Written informed consent to participate in this study was provided by the participants.

## Author contributions

HC: Conceptualization, Writing – original draft. CL: Investigation, Writing – review & editing. AZ: Software, Writing – review & editing. W-QL: Data curation, Writing – review & editing. KW: Methodology, Writing – review & editing. W-KC: Supervision, Writing – review & editing.
